# Analysis of Digital Documentation Speed and Sequence Using Digital Paper and Pen Technology During the Refugee Crisis in Europe: Content Analysis

**DOI:** 10.2196/13516

**Published:** 2019-08-19

**Authors:** Kai Kehe, Roland Girgensohn, Walter Swoboda, Dan Bieler, Axel Franke, Matthias Helm, Martin Kulla, Kerstin Luepke, Thomas Morwinsky, Markus Blätzinger, Katalyn Rossmann

**Affiliations:** 1 Department F Bundeswehr Medical Academy Munich Germany; 2 Walther Straub Institute of Pharmacology and Toxicology Ludwig-Maximilians-University of Munich Munich Germany; 3 Department E Bundeswehr Medical Academy Munich Germany; 4 University of Applied Science Neu-Ulm Neu-Ulm Germany; 5 Department of Trauma Surgery and Orthopaedics Reconstructive Surgery, Hand Surgery, Burn Medicine German Armed Forces Central Hospital Koblenz Koblenz Germany; 6 Department of Anaesthesiology, Intensive Care, Emergency Medicine and Pain Medicine German Armed Forces Hospital Ulm Germany; 7 Department VI-2.2 Bundeswehr Medical Service Headquarters Munich Germany; 8 Akademie der Unfallchirurgie Munich Germany

**Keywords:** digital documentation, digital pen, digital paper, refugee camp, refugee crisis, Europe, Germany, epidemiology

## Abstract

**Background:**

The Syria crisis has forced more than 4 million people to leave their homeland. As a result, in 2016, an overwhelming number of refugees reached Germany. In response to this, it was of utmost importance to set up refugee camps and to provide humanitarian aid, but a health surveillance system was also implemented in order to obtain rapid information about emerging diseases.

**Objective:**

The present study describes the effects of using digital paper and pen (DPP) technology on the speed, sequence, and behavior of epidemiological documentation in a refugee camp.

**Methods:**

DPP technology was used to examine documentation speed, sequence, and behavior. The data log of the digital pens used to fill in the documentation was analyzed, and each pen stroke in a field was recorded using a timestamp. Documentation time was the difference between first and last stroke on the paper, which includes clinical examination and translation.

**Results:**

For three months, 495 data sets were recorded. After corrections had been made, 421 data sets were considered valid and subjected to further analysis. The median documentation time was 41:41 min (interquartile range 29:54 min; mean 45:02 min; SD 22:28 min). The documentation of vital signs ended up having the strongest effect on the overall time of documentation. Furthermore, filling in the free-text field clinical findings or therapy or measures required the most time (mean 16:49 min; SD 20:32 min). Analysis of the documentation sequence revealed that the final step of coding the diagnosis was a time-consuming step that took place once the form had been completed.

**Conclusions:**

We concluded that medical documentation using DPP technology leads to both an increase in documentation speed and data quality through the compliance of the data recorders who regard the tool to be convenient in everyday routine. Further analysis of more data sets will allow optimization of the documentation form used. Thus, DPP technology is an effective tool for the medical documentation process in refugee camps.

## Introduction

Since 2010, the geopolitical situation in the Middle East, as well as in Africa, has become more and more fragile. An increasing number of refugees, asylum seekers and migrants are attempting to reach the European Union [1]. A refugee is defined as a person fleeing persecution or lack of protection [2], and the protection of refugees is set out in the 1951 Refugee Convention and its 1967 Protocol [3]. An asylum seeker is someone who is claiming refugee status but whose status has not yet been determined; however, the term migrant is loosely defined. United Nations High Commissioner for Refugees (UNHCR) defines migrants as people who (temporarily) change their place of residence to another country or administrative unit [4]. Europe is still facing a huge influx of refugees and migrants [5], with the Syria crisis in particular forcing more than 4 million people to leave their homeland [6]. In 2015, approximately 1 million refugees entered the European Union [7].

Refugees come from devastated countries with poor health standards, and they cross several borders and live under poor conditions during their journey. Due to Europe’s overcrowded reception stations, with their limited sanitary resources, there is a constant risk of the spread of communicable diseases. Thus, these stations pose a substantial health risk to Europe that needs to be controlled. European (as well as national) health security depends on individual health security to protect public health [8,9].

A medical information technology (IT) system dealing with the movements of significant numbers of patients requires several components: patient tracking, patient regulating, medical documentation management and exchange, medical asset tracking, medical capability assessment and sustainability analysis, provision of epidemiological statistics, report generation, and provision of relevant medical data [10]. The refugee crisis therefore called for the rapid establishment of a health surveillance system, and especially an early warning and detection system in order to meet minimum quality standards as defined by the criteria above. An early warning surveillance system is an effective tool to reduce outbreaks in refugee camps, with examples such as Bill Gates recommending building up such a warning and response system in the context of the Ebola crisis [11]. Syndromes definitions used in this paper were developed by the NATO Centre of Excellence for Military Medicine, Munich, and will be published elsewhere (personal communication by Katalyn Rossmann, 18.02.2018).

A syndromic surveillance system was implemented in Italy during a similar situation. However, documentation involved a purely paper-based system from which reporting data had to be extracted manually, and reports were being sent by e-mail to the Italian Ministry of Health or the National Centre for Epidemiology, Surveillance and Health Promotion of the National Institute of Health (CNESPS-ISS). Napoli et al concluded that this system had limitations regarding data quality and the rapid preparation of reports, so a web-based system was recommended to overcome these obstacles [12].

Other studies have demonstrated the effectiveness of web-based surveillance systems such as SurvNet developed by the Robert Koch-Institute [13] or the QSurveillance system used by Public Health England in the United Kingdom [14].

Adequate documentation is essential to ensure proper medical records and information flow between medical facilities. Furthermore, these data can be used for quality management systems and for health surveillance. Good data quality is essential for a scientific analysis of medical data but meeting these quality requirements poses a challenge for frequently used paper-based documentation systems.

A transfer from analogous data to digital data normally requires an additional step after patient contact, which is the manual typing of data into a local computer system, or web-based front-end of a client, or server-driven hospital information system, or a registry. This additional step has a negative effect on data quality, performance and process costs, as it is both time consuming and additional effort for the data recorder, without any instantaneously recognizable advantages [15]. It also introduces an additional possibility for data errors.

To improve the speed of documentation and to reduce errors, the intuitive layout of the documentation sheet is crucial [16]. However, paper-based documentation does not allow an analysis of the speed and sequence of filling the form. Digital paper and pen (DPP) technology aims to overcome this shortcoming, and several studies have been published demonstrating the effectiveness of DPP technology in various settings.

In Germany, DPP technology has been tested in several studies, especially in air rescue and emergency room data recording. The technology allowed medical documentation to be carried out with an electronic (digital) pen on special paper. After completing the form, the data sets from the pen were transferred to a computer system, approved by the physician or qualified medical personnel, and then stored in a hospital information system. The digital pen provided a timestamp for each data entry field on the form, which allowed in-depth analysis of the process of completing the form. On average, the approval time was less than 2 minutes. Data quality for core data was greater than 95% and superior to handwritten documentation, and both checkbox and numerical data fields were correctly recorded at 99.8% [15-18].

In addition to civilian organizations, the Bundeswehr also developed a near real-time syndromic surveillance system, known as the Visitor and Immigrant Health Surveillance and Information Tool (VISIT). This system was implemented at the refugee camp in Bad Fallingbostel (Lower Saxony, Germany), which was the first time that DPP technology for patient recording and automated epidemiological data collection for an early warning system had been used.

The aim of the present study was to analyze the data sets concerning the speed and sequence of medical documentation to improve data quality, process performance, and costs.

## Methods

### Digital Paper and Pen Technology

#### Digital Pen

The digital pen was purchased from Diagramm Halbach (Schwerte, Germany), and the technology was developed by Anoto (Lund, Sweden). The digital pen consists of a standard ink cartridge, a front-facing infrared camera, a microprocessor, a storage chip, Bluetooth connectivity and a universal serial bus (USB) interface. A pressure sensor starts the data-capture process. The front camera is located under the ink cartridge and captures 50 frames per second with a timestamp, while the positioning of the pen on the paper is calculated by a special pattern of dots on the paper. Less than 2 square millimeters are needed for the correct calculation of the position, and every stroke is stored in the pen. The storage capacity of each pen is up to 50 filed protocols, which was never exceeded during the field trial. A battery of a charged pen lasts up to 15 h. Finally, every pen has a unique ID.

#### Digital Paper

All digital paper has a slight (almost invisible) pattern of black dots on it. This pattern allows the digital pen to calculate the coordinates of the pen tip on the paper as well as the unique ID of the paper. Thus, digital paper is an essential part of the digital process. The VISIT forms were printed with this pattern. The position of each data field was encoded with dotforms software (Diagramm Halbach, Germany).

#### Data Transfer and Validation

A DPP docking station was connected to a standard personal computer (PC) via a USB cable. After connecting the digital pen to this station, the data were transferred to the PC. Once the raw data had been transferred to a notebook, a lookup table matched the recorded coordinates with the relevant field. In free-text fields (eg, blood pressure), optical character recognition (OCR) of handwriting was used to create a digital entry. Every marked field and OCR result was recorded in an XML-file which allowed further analysis, and then all the datasets were exported as XML-files. DPP technology could store the time of documentation for every sheet and pen, thus building up a database for further analysis of the medical documentation process.

### Data Collection

The data were collected over 3 months, from May 2, 2016 to August 2, 2016, during the field trial of the VISIT project at the refugee center in Bad Fallingbostel. The center is composed of two camps with a total capacity of 2000 persons. The German Red Cross (DRK) and the Johanniter-Unfallhilfe (JUH) run each camp, respectively. After study approval, the medical personnel in both camps were quickly trained to use the DPP technology. The study group consisted of two reception lines which were on duty every day, and during the study period a total of 495 data sets were collected. The Bundeswehr provided support for both camps with regard to medical personnel and medical documentation.

Medical personnel filled in the standardized protocol during medical examinations, and signs and symptoms, the clinical diagnosis, and sociodemographic characteristics (eg, year of birth, nationality and gender) were collected from the patients. The DPP technology was used consistently; however, personal information was not stored in the DPP system by paper design (no digital pattern) in order to protect patient privacy. Thus, only anonymous data were processed.

For each patient, one protocol (sheet of digital paper) was completed. Each protocol consisted of 277 fields into which data could be entered (either text or symbols, eg, check marks). Of course, for any given patient, many of the possible fields were left blank as they did not pertain to that specific patient.

At the end of each day, collected medical data were then transmitted, via a protected transfer, to central servers in Koblenz and Ulm, Germany. A data subset concerning syndromic surveillance was encrypted and transmitted to the Bundeswehr University in Munich and to the Deployment Health Surveillance Capability (DHSC) for further analysis by the specialists of the Bundeswehr Medical Service Headquarters as well as the DHSC. The DHSC provides epidemiological surveillance for the North Atlantic Treaty Organization (NATO) deployment areas and is a satellite branch of the NATO Centre of Excellence for Military Medicine (MILMED COE) in Budapest, Hungary.

### Process of Medical Documentation

Documentation time was the difference between first and last stroke on the paper, which includes clinical examination and translation. In the initial phase of the study, the medical documentation process was not uniform. In one medical treatment facility, only the documentation sheet accompanied the patient. This resulted in entries being produced by different pens on one documentation sheet with multiple XML-files. As for all the other documentation processes, a unique digital pen and a documentation sheet accompanied the patient, resulting in a single XML-file per person.

### Data Set

The data set was developed by the DHSC and the Bundeswehr Medical Service Headquarters. The implemented data set is a subset of the German national data set regarding anything emergency department–related that is relevant for primary health care [19]. This collection was complemented by adapting the syndrome-specific parameter, resulting in a total of 64 sets of data. For further statistical analysis, the diagnosis was matched to the 10^th^ revision of the International Statistical Classification of Diseases and Related Health Problems (ICD-10) code, according to the World Health Organization (WHO) list.

The VISIT protocol sheets are available upon request from the authors.

### Analysis of Time Stamps

**Figure 1:**
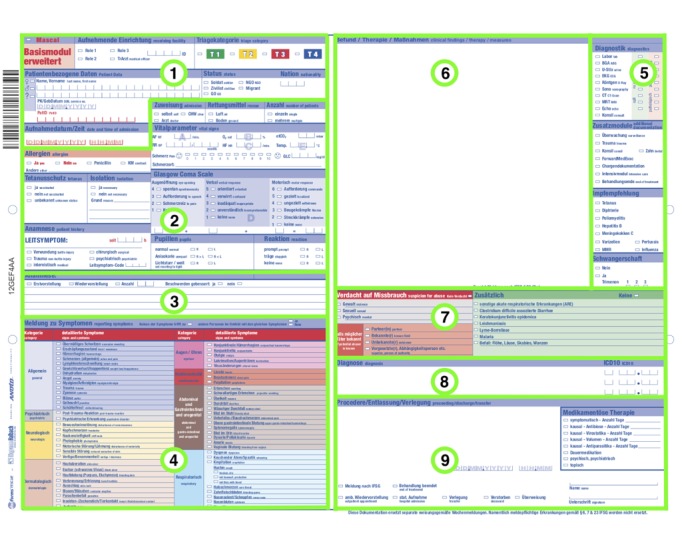
Form for essential documentation. Fields are clustered into nine groups. Group 1: patient information. Group 2: patient history and vital data. Group 3: free text patient history. Group 4: symptoms and signs. Group 5: diagnostic and vaccination recommendation. Group 6: free-text documentation. Group 7: sexual abuse and rare diseases. Group 8: free text diagnosis with coding. Group 9: proceeding, recommendation, and transfer.

With the goal of optimizing the processing time, we asked which fields had the strongest effect on total examination time for a patient. For this analysis, we grouped the 277 fields into 70 categories, pooling fields covering different aspects of the same topic into one category. For example, the category “symptoms eye/ear” consists of five fields covering symptoms from conjunctival hemorrhaging to otalgia (see upper right corner of section 4 in Figure 1). Then, a Mann-Whitney *U* test was conducted to compare the documentation time of reports where a specific category was completed (at least one of the fields filled out) with those where this specific category was empty.

For a more detailed analysis, we also asked which fields required the most time for completion. This analysis is possible since, for each field, the time stamps of the first and the last entry into the field are stored. This is not corrected for cases where the examiner goes back and forth between fields. If, for example, the examiner starts filling out field A, then makes entries into field B, and then finally goes back to field A and completes it, then the time stamp would count the whole time from the first change in field A to the last. In this sense, our analysis captures those fields which are the most complicated to fill out.

Finally, the design of the documentation sheet determines the speed at which the form can be completed. As the digital pen stores a timestamp for each entry, the sequence of documentation can be analyzed. The total time of documentation for each sheet was set at 1, so each timestamp was normalized and expressed as a value between 0 and 1. The fields of the documentation sheet were then grouped into nine sections (Figure 1). For each sheet, the normalized time of the first entry into each section was noted, thus, the order in which the sections were worked on can be analyzed. For example, the median of these normalized times for a section expresses the time point (relative to the total time needed for the full medical examination) where half of the examiners have started on that section.

### Statistical Analysis

The XML files were imported into Microsoft Excel (version 15.29) and prepared for further analysis. Statistical analysis was performed using standard statistical software (SPSS version 24 for Windows), depicted as box-and-whisker plots. The effect of different pens on time of documentation was analyzed with the Mann-Whitney *U* test.

### Ethical Approval

Prior to the investigation, ethical clearance was obtained from the Ethics Commission of the Ludwig-Maximilians-University of Munich (143-16). Verbal informed consent was received from the refugees.

## Results

### Effect of Optimizing Digital Pen and Paper Handling

Altogether, 495 data sets (XML files of individual documentation sheets, each documenting one patient) were generated. However, after an initial phase of training personnel and optimizing the documentation process, the procedure was adapted. During the initial phase, digital pens were available at each station. This ended up causing multiple entries per sheet being made with different digital pens, which caused problems with respect to merging data sets and data integrity. This technical problem was solved when the digital paper and pen accompanied the patient during the whole medical examination process. As a result, 74 data sets, generated during this initial phase with more than one digital pen ID, were excluded from further analysis, leaving 421 data sets to be analyzed.

### Time of Documentation

**Figure 2:**
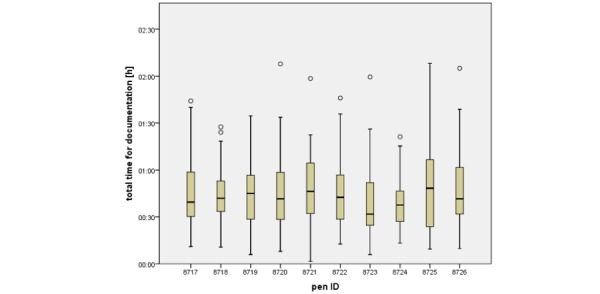
Time of documentation. The median time of documentation for each pen was calculated. Boxplots show the lowest and highest values of time of documentation, a quantitative measure for the length of the medical process. Boxes represent the inter-quartile range (25th to 75th percentile), and whiskers indicate the minimum and maximum of the data except for outliers (shown as circles). The thick horizontal line within each box represents the median. ID: identification.

The total time of documentation (time difference between first and last stroke on a documentation sheet) ranged between about 90 seconds and over 2 hours, with a median of 41:41 minutes and an interquartile range (IQR) of 29:40 minutes (mean 45:02; SD 22:28 min). The distribution of these times is skewed to the right, and the Kolmogorov-Smirnov test rejects the null hypothesis of the normal distribution (*P*=.03). The median time of documentation for individual pens varied from 31:44 min to 48:20 min, and the number of completed forms per pen ranged between 28-62. A summary of documentation times per pen is presented in Figure 2; however, the time of documentation for the different pens showed no significant difference, according to the Kruskal-Wallis test *(P*=.54).

### Fields with a Significant Effect on the Speed of Documentation

Overall, 21 out of 70 categories have a significant effect on documentation time. The data are shown in Table 1. The documentation of vital signs (eg, blood pressure, blood oxygenation, heart rate, temperature, and blood glucose) had the highest impact on documentation time, with 5 out of the 21 significant categories belonging to this group. Additionally, anything connected to the calculation of the Glasgow Coma Scale also had a significant effect on overall documentation time. Interestingly, sheets where eye, ear or dermatological symptoms were documented had a significantly shorter completion time. A Mann-Whitney *U* test was conducted to compare the documentation time of reports with entries in a category and reports without entries in the same category. Significant effects were considered where *P*<.05.

**Table 1:** List of field categories with significant effect on time of documentation.

Categories with significant effect		Reports, n	Average difference, min	*P* value^a^	
Blood pressure		269	15:49.9	<.001	
Oxygen saturation		249	15:23.9	<.001	
Heart rate		250	14:35.2	<.001	
Temperature		218	06:11.0	<.001	
**ICD^b^ code**					
	Field 2	221	08:14.5	<.001
	Field 3	95	04:38.8	.03
Urine stick		75	09:41.3	<.001	
Patient history		190	07:40.4	.001	
**Glasgow Coma Scale**				
	Eye	150	04:55.6	.02
	Motor	150	04:55.6	.02
	Verbal	150	04:55.6	.02
	Sum	88	08:14.2	.002
**Symptoms**					
	Eye or ear	35	–08:10.0	.008
	Other person with same symptoms	79	–05:25.4	.008
	Dermatological	19	–08:12.9	.03
	Cardiovascular	4	21:11.4	.045
Blood glucose		39	08:16.5	.03	
**Medication**					
	Symptomatic or acute	162	05:03.1	.013
	Long-term	113	06:07.6	.02
	Antibiotics	67	–05:12.3	.04
ECG^c^		9	17:02.5	.04	

^a^Value was calculated using the *U* test.

^b^ICD: International Statistical Classification of Diseases and Related Health Problems.

^c^ECG: electrocardiogram.

### Time Needed per Field

For each field, the time needed to fill it was calculated. Table 2 shows the nine fields with the highest median times (4 seconds or more) for completion. The large free-text field called clinical findings, or therapy, or measures, needed the most time (median 8:06 min; IQR 23:56 min). The next four most time-consuming fields were also free-text fields: patient history, proceeding, diagnosis and discharge.

### Sequence of Documentation

**Figure 3:**
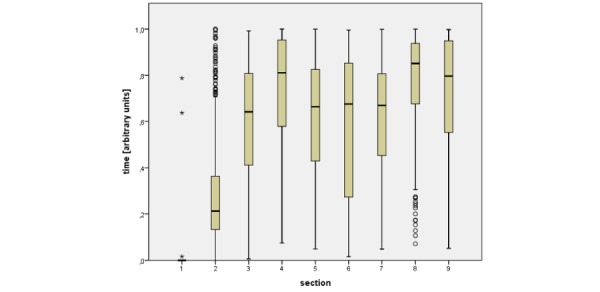
Sequence of documentation. Fields are clustered into nine sections. Section 1: patient information. Section 2: patient history and vital signs. Section 3: free text patient history. Section 4: symptoms and signs. Section 5: diagnostics and vaccination recommendation. Section 6: free-text documentation. Section 7: sexual abuse and additional rare diseases. Section 8: free text diagnosis with coding. Section 9: proceeding, recommendation and transfer.

The data presented in Figure 3 shows the sequence of documentation. Almost every medical examiner started with the first section (patient personal data and manner of transfer to the medical center). Section 2 (patient vital signs and medical history) usually came next. Following that, the diagnostic sections 3, 5, 6, and 7 were started more or less interchangeably and at the same time. Interestingly, half of the medical examiners made their first entry into section 4 (symptoms) only after the other diagnostic sections, namely at roughly the same time as the concluding sections 8 (diagnosis with ICD code; this was often the last section to be started) and 9 (further proceeding, discharge or transfer). It should be noted, however, that these times (except for section 1) displayed large variances as seen in Figure 3.

**Table 2:** Time needed per field. The table lists all fields with a median documentation time of 4 seconds or more (N=421).

Field	n	Median (IQR^a^), min	Mean (SD), min
Clinical findings	412	8:06 (23:56)	16:49 (20:33)
Patient history	407	2:07 (5:39)	4:35 (5:59)
Proceeding	293	1:06 (4:06)	3:59 (6:28)
Diagnosis	409	0:26 (2:35)	3:04 (6:14)
Discharge	113	0:13 (0:18)	1:01 (3:16)
Transfer	50	0:06 (0:12)	0:21 (0:57)
Date of admission	418	0:06 (0:02)	0:07 (0:06)
Allergies (other)	17	0:05 (0:05)	0:09 (0:12)
Patient ID^b^	416	0:04 (0:02)	0:11 (1:49)

^a^IQR: interquartile range.

^b^ID: identification.

### Number of Data entries per Category

Data are shown in Table 3. The categories more frequently completed (i.e. more than 90%) were patient data (99.8%), receiving facility (99.5%), time of admission (99.3%), proceeding date (99.0%), status (98.1%), first ICD-10 code (97.1%), patient history (96.2%), nationality (96.0%) and self-admission (95.2%). 17 of the 70 categories had no entry in all the analyzed data sets; 11 had only one entry.

**Table 3:** Number of data sets with entries in the field categories. Table shows categories with entries in more than 95% of data sets as well as categories which have been edited at most once (N=421).

Category		n (%)	
Patient related data		420 (99.8)	
Reception facility		419 (99.5)	
Recording date		418 (99.3)	
Procedure date		417 (99.0)	
Status		413 (98.1)	
ICD^a^ code 1		409 (97.1)	
Patient history (details)		405 (96.2)	
Nationality		404 (96.0)	
Self-assigning		401 (95.2)	
Allergy		1 (0.2)	
Number of patients		1 (0.2)	
**Diagnostics**				
	CT^b^ scan	1 (0.2)
	Consult	1 (0.2)
	ABG^c^	0 (0)
	Echocardiogram	0 (0)
	MRI^d^	0 (0)
	Sonography	0 (0)
	X-ray	0 (0)
**Vaccination**				
	Meningococcus	1 (0.2)
	Measles, mumps, rubella	1 (0.2)
	Diphtheria	0 (0)
	Hepatitis B	0 (0)
	Influenza	0 (0)
	Pertussis	0 (0)
	Poliomyelitis	0 (0)
	Tetanus	0 (0)
	Varicella	0 (0)
**Procedure**				
	Transfer	1 (0.2)
	Dead	1 (0.2)
Suspicion of sexual abuse		1 (0.2)	
Vital CO_2_^e^ parameter		1 (0.2)	
Consultation		1 (0.2)	
MASCAL^f^		0 (0)	
**Medication**				
	Virostatic agents	0 (0)
	Volume	0 (0)
Rescue		0 (0)	
Triage category		0 (0)	

^a^ICD: International Statistical Classification of Diseases and Related Health Problems.

^b^CT: computed tomography.

^c^ABG: arterial blood gas.

^d^MRI: magnetic resonance imaging.

^e^CO_2_: carbon dioxide.

^f^MASCAL: mass casualties.

## Discussion

### Principal Findings

Taking handwritten notes is one of the least technical ways to collect medical data. Generations of medical doctors have been trained to use paper-based documentation and to note their clinical findings [20], however, the enormous medicolegal need for documentation poses a considerable challenge [21]. Additionally, paper-based documentation should be phased out and instead be digitized for long-term storage, integration into hospital information systems, and for further analysis with respect to quality management [22].

The speed of handwriting is limited and slows down the documentation process even further, as the use of other devices (eg, smartphones, typewriting or keyboard-based typing on personal computers) increases [23]. Therefore, forms are needed to ensure both reliable data collection as well as to speed up the process of filling them in through structured protocols. To overcome the obstacles of paper-based documentation, new technologies are emerging to support the documentation process. Speech recognition [24,25], automated data recording [26,27], and DPP technology [14] are examples of this. Handwriting recognition is improving and being implemented in standard office software suites, but it can still lead to errors [28,29]. The same holds true for speech recognition, although further training is needed as well as a relatively silent ambience around the user [23]. Voice recognition technology often shows no advantage over manual transcription [30]; however, the rise of artificial intelligence (AI) or deep machine learning in combination with voice recognition is promising new technologies to further speed up the documentation process.

In the case of mass casualties during a crisis, it is crucial to increase the speed of medical documentation without losing quality. Processing speed and data quality should be as high as possible to cope with a large number of patient casualties [31]. In this context, valid data for health surveillance are of utmost importance [11], but the recorded information is also of interest for evaluating complex interventions [32]. The data imported into automated health surveillance systems should be as simple as possible, and in the best case it should be an automatic byproduct of the legally required patient documentation. DPP technology has proven to be a good combination of both paper-based records and electronic documentation [14,33].

No statistical difference was identified between the digital pens used. Thus, both reception lines worked equally effectively. Apart from the first week, use of the technology was very reliable. The pen strokes from the 421 collected data sets allowed a reconstruction of the sequence in which the fields were filled in the form. The graphical representation of the sequence showed that the design of the report seems to be appropriate. As stated above, the diagnostic sections were recorded earlier than the final diagnosis field, including the ICD-10 code that was often the last field to be worked on. A request submitted to the medical documentation team revealed that matching the diagnosis to the ICD-10 code required additional time, and thus the ICD-10 code was added as the last step of medical documentation. This personal observation is backed up by the fact that the step to match the diagnosis with the ICD code had a significant impact on overall documentation time. Additionally, the diagnosis and proceedings fields were among the five fields with the longest documentation time.

Another time-consuming block involved everything related to the examination of the patient. The clinical findings, or therapy, or measures free-text field required the most time for documentation per field. Furthermore, the recording of vital signs had the highest impact on documentation time. A total of 98% of the clinical findings, or therapy, or measures field, and approximately 60% of the significant vital sign fields, were filled in. Thus, the optimization of this step has great time-saving potential.

The fields with the most data entries are assumed to be more relevant. By contrast, fields with little or no data entry must be considered less important and are thus probably dispensable in further studies. The recorded pen strokes clearly showed that data sets were recorded during the patient examination process and not afterwards. It has been shown in previous studies that the completeness of data sets is superior to any kind of retrospective documentation [34,35]. Furthermore, total documentation time is reduced using DPP technology compared to paper-based documentation, where secondary data entry in a computer system is necessary and errors may occur. The time required to transfer all the data sets from a pen is less than 10 min. Given that 30 min per form are needed for secondary data entry, the time saved is estimated to be 210 hours in total, which is approximately 26 days of work, or more than one month, for one documentation assistant. Thus, DPP technology has a return on investment that will increase the longer this technology is used. Additionally, the time-saving effect in epidemiological surveillance is crucial, as with DPP technology more surveillance reports can be generated in less time compared to traditional paper-based systems [32].

The strength of this study lies in its description of how DPP technology can be used to analyze the speed of medical documentation, as well as the behavior of the medical documentation team [36]. In particular, it was possible to conduct detailed analysis of the documentation sequence and speed with regard to the form used. Furthermore, the study highlighted the possibility of training medical personnel very quickly to use DPP technology. However, the main limitation of this study lies in the limited timeframe of data capture. It could be seen as a longitudinal cross-sectional study with the inherent weaknesses of this study design, but to acquire more valid data, it is necessary to analyze more data sets. Furthermore, it may be of interest to see whether a learning curve is detectable as users adapt to this new technology. A cohort design is likely to produce more reliable and precise data regarding these questions.

### Conclusion

We conclude that medical documentation using DPP technology leads to an increase in documentation speed and quality. Further analysis of more data sets will allow optimization of the documentation form used. DPP technology is an effective tool for the medical documentation process in refugee camps.

## Abbreviations

AI: artificial intelligence
CNESPS-ISS: National Centre for Epidemiology, Surveillance and Health Promotion of the National Institute of Health
DHSC: Deployment Health Surveillance Capability
DPP: digital pen and paper
DRK: German Red Cross
ICD-10: International Statistical Classification of Diseases and Related Health Problems–10th revision
IQR: interquartile range
IT: information technology
JUH: Johanniter-Unfallhilfe
MILMED COE: NATO Centre of Excellence for Military Medicine
NATO: North Atlantic Treaty Organization
OCR: optical character recognition
PC: personal computer
UNHCR: United Nations High Commissioner for Refugees
USB: universal serial bus
VISIT: Visitor and Immigrant Health Surveillance and Information Tool
WHO: World Health Organization
